# Supplementary figures and images for: Diverse lineages of pathogenic Leptospira species are widespread in the environment in Puerto Rico, USA
Source: PLoS Negl Trop Dis. 2022 May 18;16(5):e0009959. doi: 10.1371/journal.pntd.0009959 (PMC9154103; doi:10.1371/journal.pntd.0009959)

- ✦ Soil Sample
- ✦ Water Sample

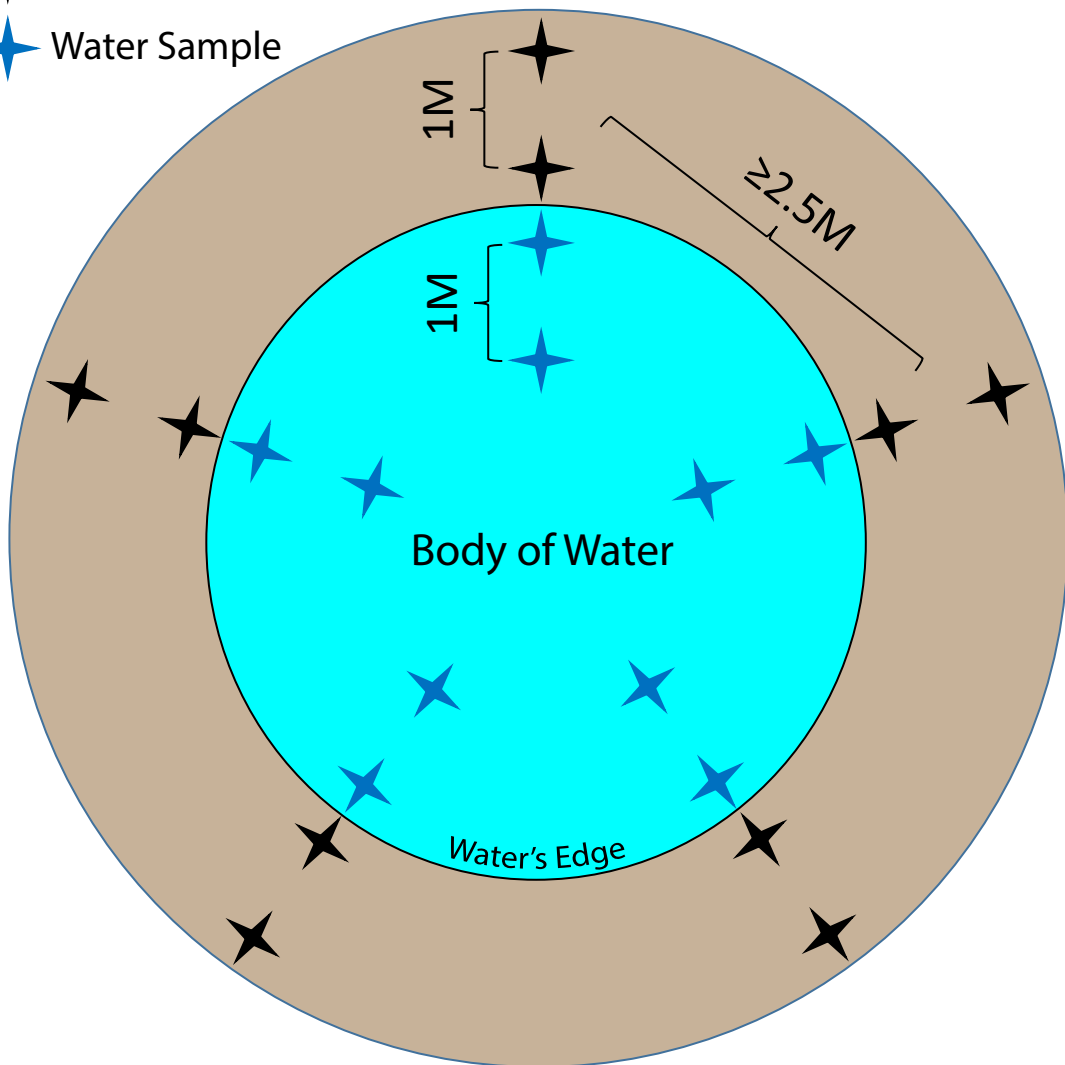

Supplement: S1 Fig — Black and blue stars represent soil and water samples, respectively. Transects were a minimum of 2.5 meters apart. This sampling design was also applied in a linear fashion for the sampling of rivers and streams. (PDF) [file pntd.0009959.s001.pdf]

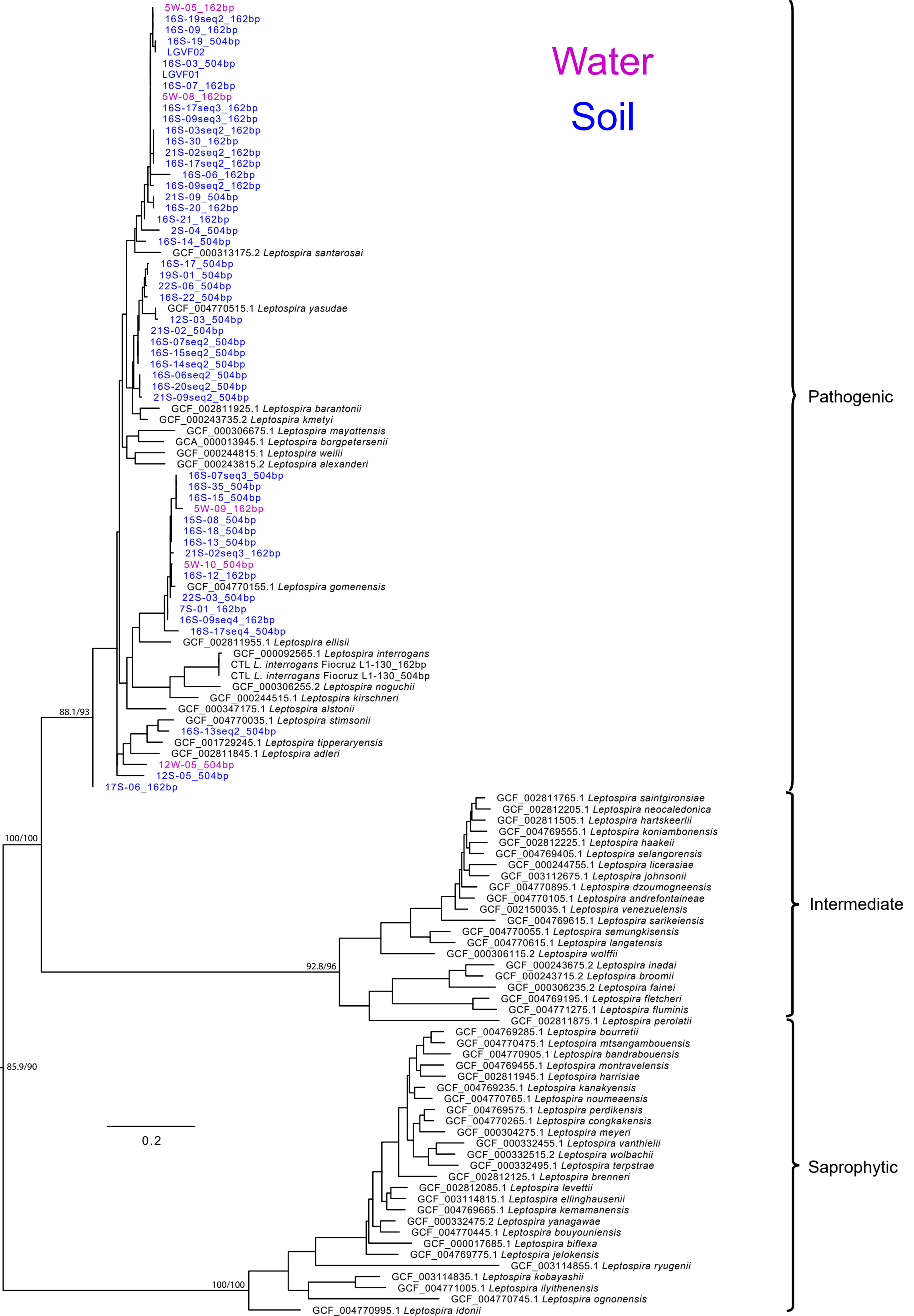

Supplement: S3 Fig — Sequences generated from soil and water samples collected in Puerto Rico are in blue or pink text, the novel pathogenic Puerto Rico isolates from soil are also included; reference sequences for pathogenic, intermediate, and saprophytic Leptospira are in black. Bootstrap/aLRT support values are indicated on branch nodes. (PDF) [file pntd.0009959.s003.pdf]

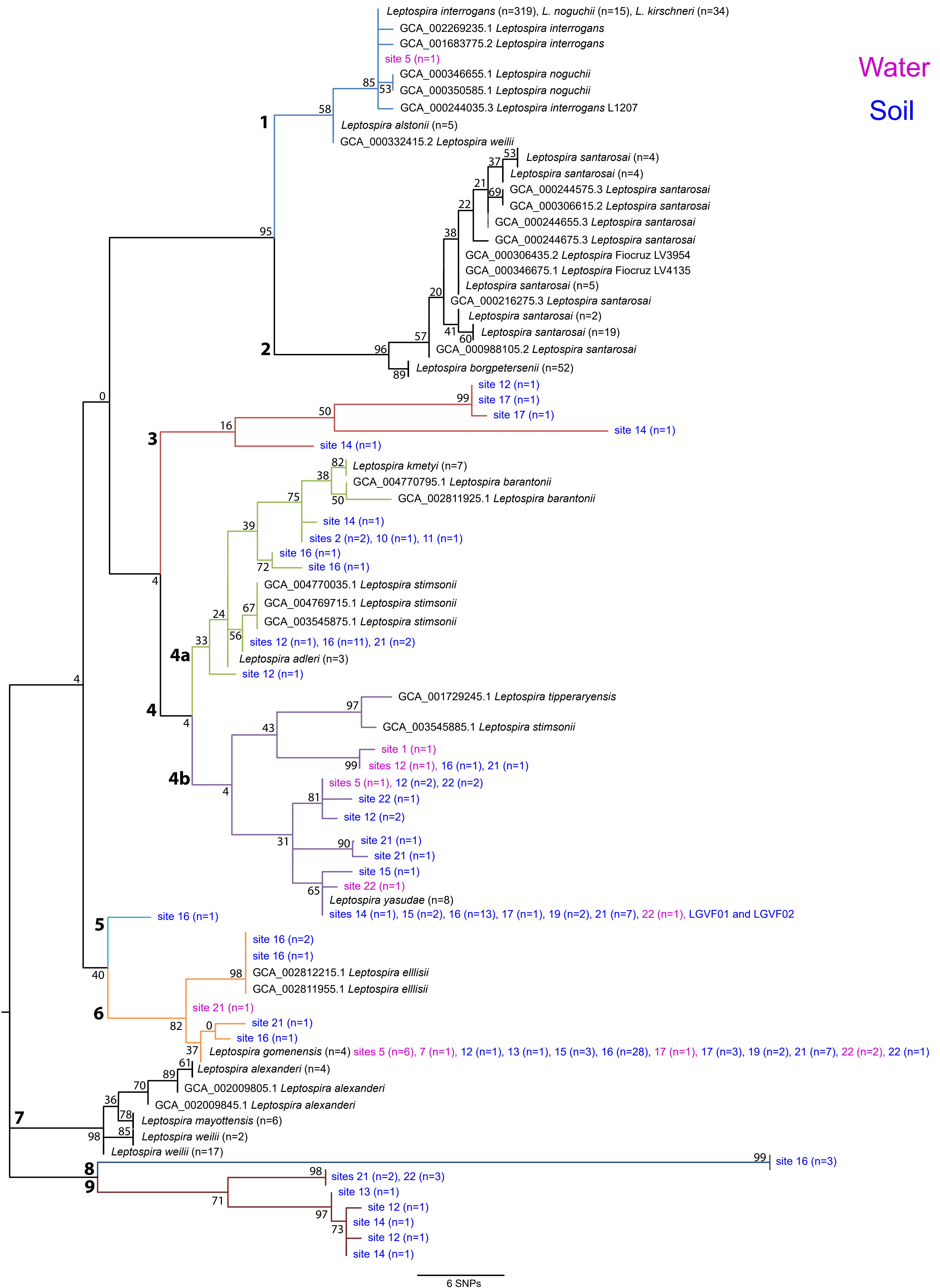

Supplement: S4 Fig — Sequences generated from soil (blue text) and water (pink text) samples collected in Puerto Rico are displayed. Reference sequences representing all other known pathogenic Leptospira spp. are in black. Nine major clades are represented of which seven were identified in Puerto Rico and four of those (3, 5, 8, and 9) have not been described previously. Identical genotypes were present in soil and water from Puerto Rico at two sites (17 and 21), with both occurrences representing a single genotype from clade 6 (in orange). Bootstrap values are indicated on each branch. Major clades are numbered 1 through 9 and color-coding matches Fig 2. (PDF) [file pntd.0009959.s004.pdf]

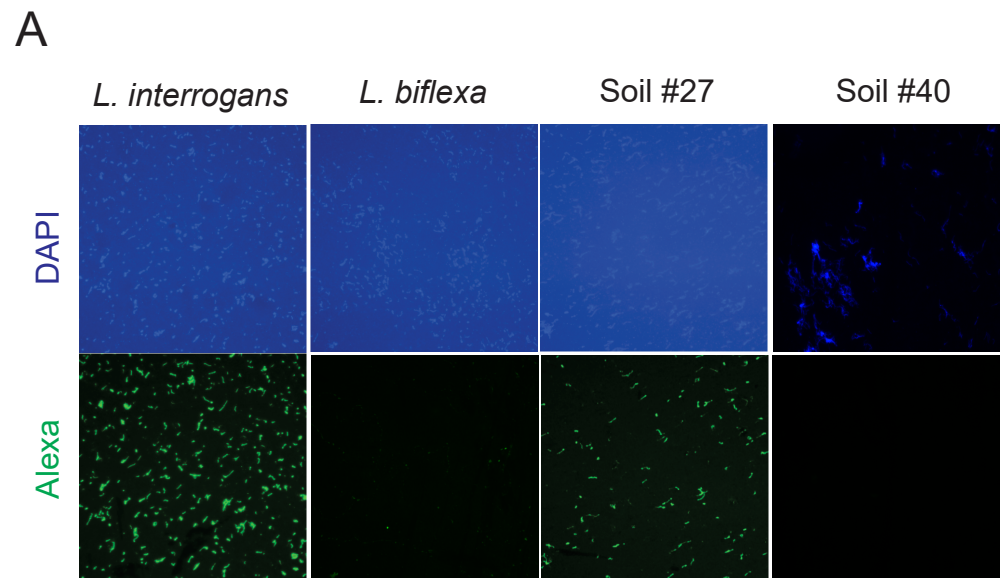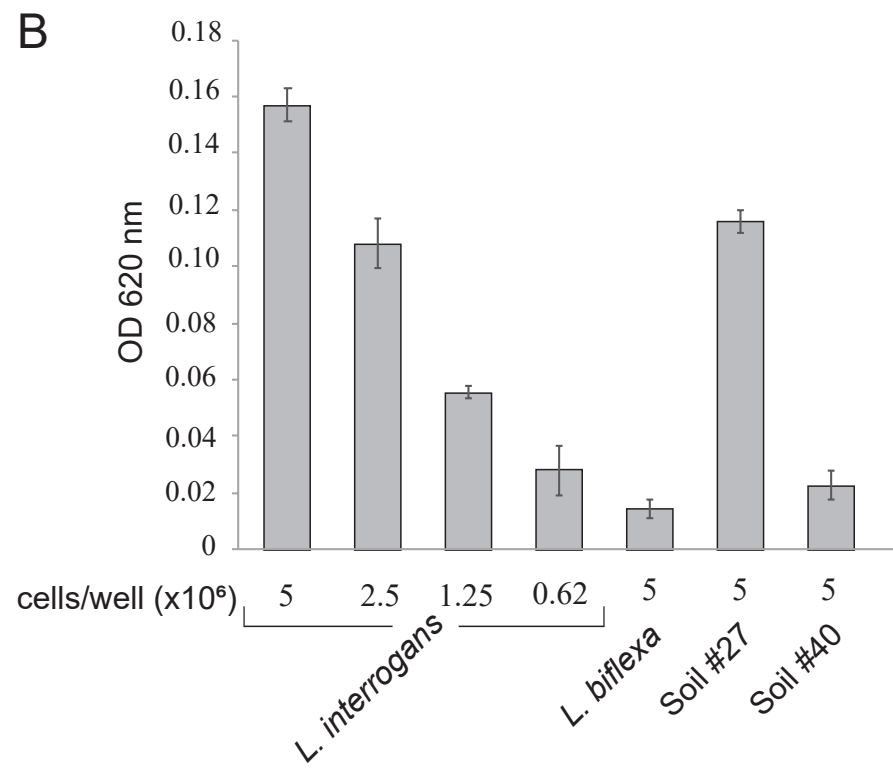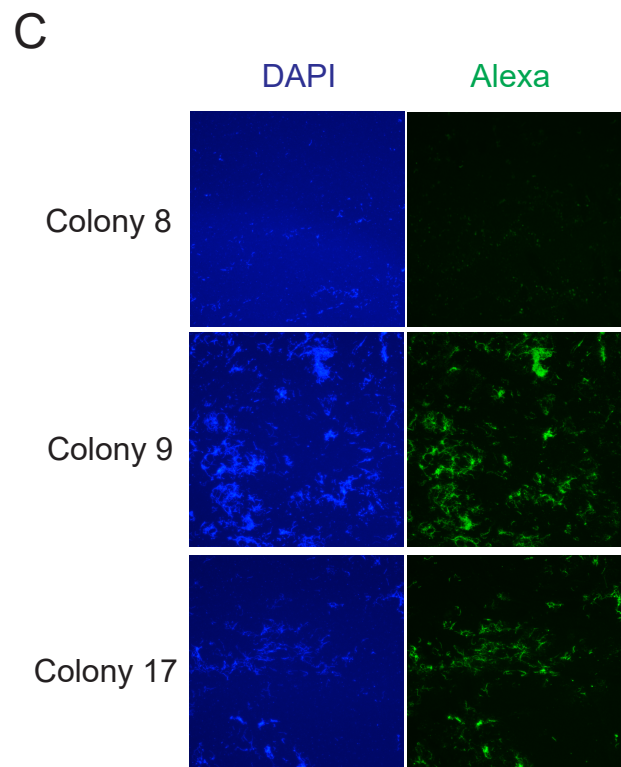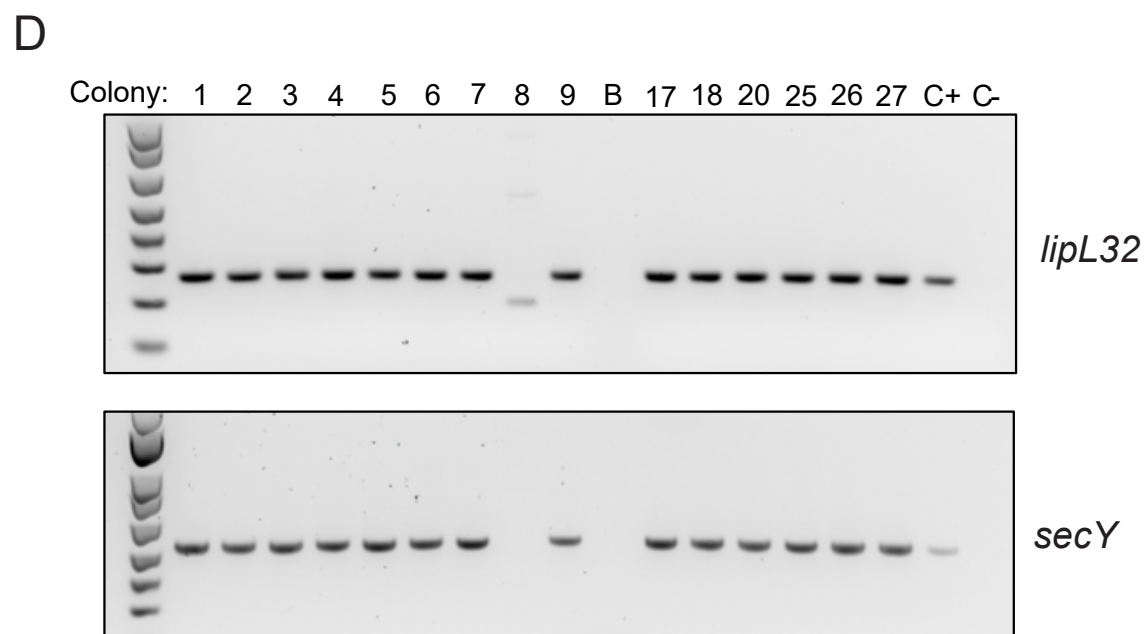

Supplement: S5 Fig — LipL32 PCR positive (Soil #27) and negative (Soil #40) samples were subjected to FAT (Panel A) and ELISA (Panel B) along with positive and negative controls (L. interrogans and L. biflexa, respectively) to assess expression of the LipL32 pathogenicity protein. Individual colonies from Soil #27 were subjected to additional FAT testing (Panel C) and confirmatory PCR (Panel D) to verify that the obtained isolates were pathogenic. (PDF) [file pntd.0009959.s005.pdf]
