# Supplementary material for: Diverse lineages of pathogenic Leptospira species are widespread in the environment in Puerto Rico, USA
Source: PLoS Negl Trop Dis. 2022 May 18;16(5):e0009959. doi: 10.1371/journal.pntd.0009959 (PMC9154103; doi:10.1371/journal.pntd.0009959)
Supplement: S2 Fig — The yellow triangles indicate sites where pathogenic Leptospira DNA was detected. Eight soil sites were positive for pathogenic Leptospira DNA, compared to two water sites; both soil and water were positive at five sites. Site 16, which was used to assess environmental persistence in soil is indicated with a red circle. This map was created using ArcGIS software by Esri. ArcGIS and ArcMap are the intellectual property of Esri and are used herein under license. Copyright Esri. All rights reserved. For more information about Esri software, please visit www.esri.com. Basemap: http://goto.arcgisonline.com/maps/Canvas/World_Light_Gray_Base. (PDF) [file pntd.0009959.s002.pdf]

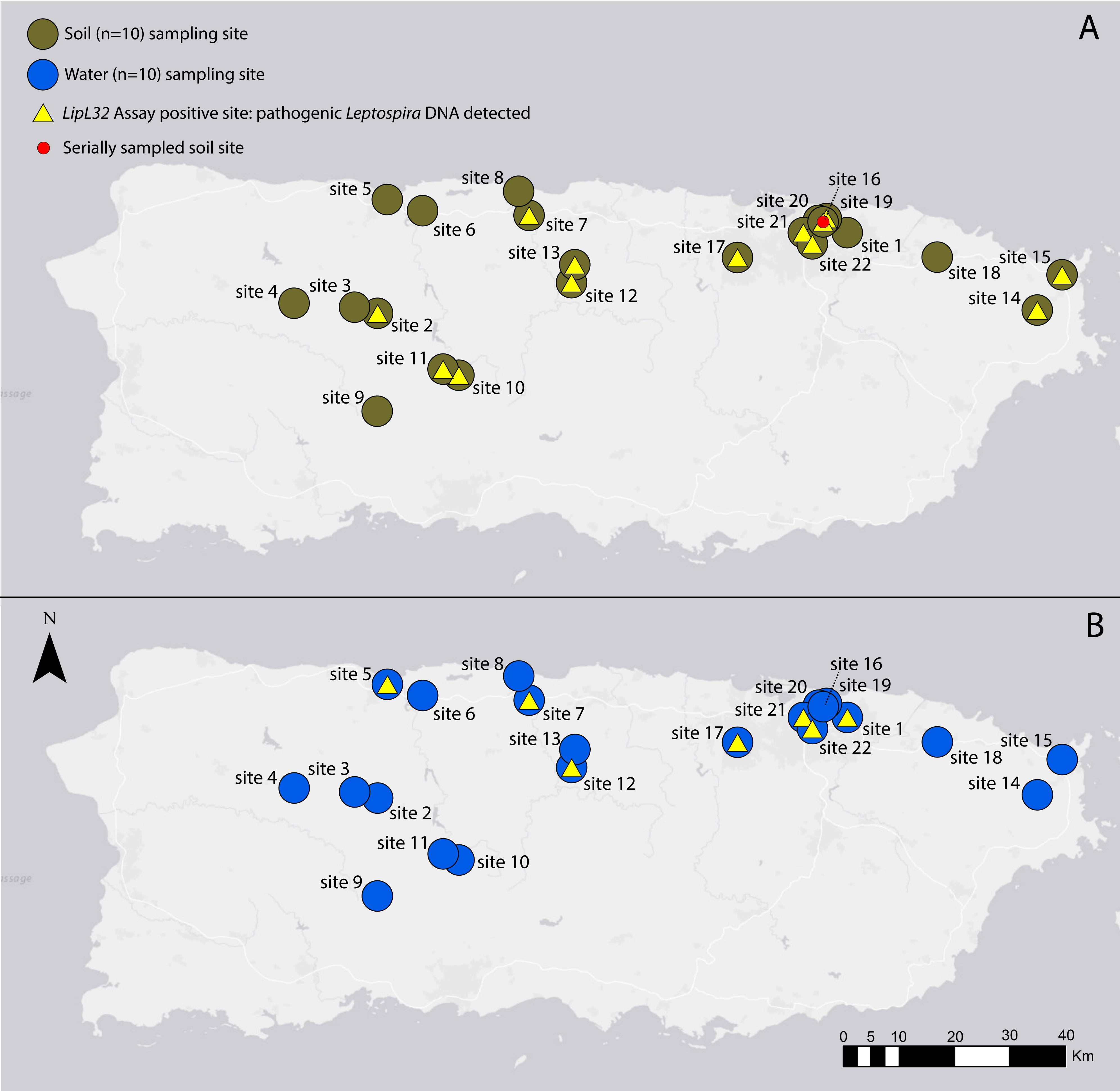

Soil (n=10) sampling site
Water (n=10) sampling site
LipL32 Assay positive site: pathogenic Leptospira DNA detected
Serially sampled soil site
A
site 1
site 2
site 3
site 4
site 5
site 6
site 7
site 8
site 9
site 10
site 11
site 12
site 13
site 14
site 15
site 16
site 17
site 18
site 19
site 20
site 21
site 22
B
N
site 1
site 2
site 3
site 4
site 5
site 6
site 7
site 8
site 9
site 10
site 11
site 12
site 13
site 14
site 15
site 16
site 17
site 18
site 19
site 20
site 21
site 22
0
5
10
20
30
40
Km
